# Barriers and facilitators to physical activity in second-generation British Indian women: A qualitative study

**DOI:** 10.1371/journal.pone.0259248

**Published:** 2021-11-03

**Authors:** Prachi Bhatnagar, Charlie Foster, Alison Shaw

**Affiliations:** 1 Nuffield Department of Population Health, University of Oxford, Oxford, United Kingdom; 2 Centre for Exercise, Nutrition and Health Sciences, School for Policy Studies, University of Bristol, Bristol, United Kingdom; 3 Institute of Social and Cultural Anthropology, University of Oxford, Oxford, United Kingdom; Public Library of Science, UNITED KINGDOM

## Abstract

**Aim:**

To understand the barriers to and motivations for physical activity among second-generation British Indian women.

**Subject:**

Approximately 50% of British South Asians are UK-born, and this group is increasing as the second-generation also have children. Previous research into the barriers to and facilitators for physical activity has focused on migrant, first-generation populations. Qualitative research is needed to understand a) how we might further reduce the gap in physical activity levels between White British women and British Indian women and b) the different approaches that may be required for different generations.

**Methods:**

Applying a socioecological model to take into account the wider social and physical contexts, we conducted semi-structured interviews with 28 Indian women living in Manchester, England. Interviews with first-generation British Indian women were also included to provide a comparator. Interviews were audio-recorded, transcribed, thematically coded and analysed using a grounded theory approach.

**Results:**

Ways of socialising, concerns over appearance while being physically active, safety concerns and prioritising educational attainment in adolescence were all described as barriers to physical activity in second-generation British Indian women. Facilitators for physical activity included acknowledging the importance of taking time out for oneself; religious beliefs and religious groups promoting activity; being prompted by family illness; positive messages in both the media and while at school, and having local facilities to use.

**Conclusions:**

Barriers to physical activity in second-generation Indian women were very similar to those already reported for White British women. Public health measures aimed at women in the general population may also positively affect second-generation Indian women. First-generation Indian women, second-generation children and Muslim women may respond better to culturally tailored interventions.

## Introduction

Reducing health inequalities is a legal requirement of the Health and Social Care Act 2012 and a key aim of the NHS five year forward view. The British Medical Association recently called for the UK government to tackle inequalities in physical activity [1] and the UK government’s recent policy paper on tackling obesity recognises the importance of physical activity and reducing inequalities [2]. Physical inactivity is linked with all-cause mortality and has consistently been shown to be a good secondary and primary preventative strategy for at least 25 chronic conditions, for example cardiovascular diseases and type-2 diabetes [3]. Higher physical activity and fitness levels also lower the risk of dying at a younger age [4]. Physical inactivity costs the National Health Service around £1 billion per year and wider society around £7.4 billion per year [5–7].

Physical inactivity is prevalent among South Asian women worldwide and in the UK, particularly with regards to leisure-time physical activity [8,9]. In England, physical activity levels among South Asians are approximately 60% lower than among White British, and lowest in South Asian women [10]; however this summative data masks the significant variation in physical activity patterns within the British South Asian population. As part of a mixed-methods project exploring variation in physical activity in UK South Asian women, we found the level and type of physical activity varied by migration generation and age [11,12]. For example, Indian women over 55 spent 76% of their physical activity doing housework, compared to 46% of Indian women aged 16 to 34 [11]. Due to the limited data available, previous research on generational differences in physical activity in South Asian women has been unable to ascertain the reasons behind these behavioural shifts, how they sit in the context of the wider social and physical environment, and what this means for targeting public health interventions.

Tailoring public health interventions ensures that the needs of local populations are met, with needs being likely to differ according to cultural, social historical and political dimensions. Tailoring public health interventions to different cultural groups is recommended to help increase the effectiveness of the interventions, with a 2013 systematic review suggesting a package of cultural adaptions and that those incorporating family values may be most effective [13]. A large UK-based project developed a tool-kit for adapting ethnic minority interventions. Areas for potential adaption of interventions were collaborative working with the target population, matching the team to the target population, endorsement from respected community members, adapting materials to reflect the target population, messages suitable for the population’s social and cultural values, and delivery of interventions in an appropriate format [14]. Appropriately targeting interventions may also increase their cost effectiveness if resources are directed towards the most influential elements. At present, we lack the necessary knowledge to reduce the gap in physical activity between second-generation South Asian women and White British women, because most research in this area has been conducted with older, first-generation women who migrated to the UK in the mid-twentieth century [12,15]. Papers published during the 1990s on ethnicity and health research discuss the problem of combining ethnic groups into broad categories such as ‘Asian’ and using country of birth as a proxy for ethnicity [16]. Using country of birth would mask the presence of South Asians born in the UK and focus research on those South Asians who, having primarily migrated during the 1950s to 1970s, would be in the middle aged and older age groups by the 1990s. Additionally, treating ethnic groups as broad categories, by definition misses or perhaps does not consider the heterogeneity within these groups. Existing research on the motivators and barriers to physical activity among UK South Asian women has mainly reported on the cultural barriers facing migrant women [9]; these include women prioritising family commitments over their own health or physical activity and considering physical activity to be unimportant [17].

As of the 2011 UK census, at least half of the UK South Asian population was born in the UK. This UK-born South Asian population is likely to have increased in number over the last decade as more South Asian children will have been born in the UK. Consequently, the existing research on motivations for and barriers to physical activity among British South Asians, which has focused on the first (migrant) generation, does not reflect the experiences of a large proportion of the UK South Asian population–namely the second-generation onwards.

The aim of this paper is to understand the barriers to and facilitators of physical activity in second-generation British Indian women, with the ultimate aim of being able to provide recommendations to tailor physical activity interventions for second-generation British Indian women.

### Theoretical framework

A socioecological model for physical activity [18] was used as the theoretical framework for this study. Socio-ecological models take many levels of influence into account, but places emphasis at the level of the physical and social environment, rather than solely on individuals [18]. Core principles of sociological models are that they recognise 1) multiple levels of influences on specific health behaviours, 2) that influences on behaviours interact across these different levels 3) that socioecological models should be behaviour-specific and 4) multi-level interventions are likely to be most effective in changing behaviour. Levels of influence cover: the intrapersonal level, such as age, sex and beliefs; the interpersonal level, consisting of friends, family, peers and colleagues; the built environment, which incorporates access to green space, recreation facilities and active travel infrastructure; the natural environment, for example the weather and topography, and finally the policy environment, which determines regulations for and investments in public infrastructure [18]. Socioecological models are particularly appropriate for studying the topic of physical activity, because physical activity is done in specific places and physical environments [18].

As a variable that is often defined in relation to another group, ethnicity can be difficult to place neatly within a socio-ecological model. However, the socio-ecological model can be useful when studying health behaviours in ethnic groups, because it helps to contextualise behaviour and reminds us that ethnic minority groups often live in the same physical and social environments as the majority population, and so may be equally affected by these. This is particularly important for South Asian groups, who tend to live in deprived, urban areas of the UK. Local environment measures such as sports facilities, perceived crime and walkability of neighbourhoods are infrequently included in papers which investigate the relationship between ethnicity and physical activity [19]. Given that the local environment is an important factor in research investigating the determinants of physical activity, approaching our study of physical activity in second-generation British Indian women through the lens of a socioecological model ensured that a wide range of physical activity determinants were considered when developing the topic guide for semi-structured interviews.

## Methods

### Study design

In this study, we report on the qualitative element of a wider mixed methods study exploring variation in physical activity within South Asian women [11,12]. We conducted semi-structured interviews with 19 second-generation Indian women. After analysing the first set of interviews, we decided to recruit some first-generation women, ideally mother-daughter pairs, to help triangulate the emerging findings and obtain the first-generation’s perspective on generational change in the physical activity of their daughters. We recruited 9 first-generation women, 4 of whom were mothers to second-generation British Indian women in the study; where a second-generation daughter did not agree to participate, we decided to include the first-generation participant as we felt she would still be able to contribute valuable perspectives on generational change in physical activity.

We defined the second-generation as those who were born in the United Kingdom (UK) or moved to the UK before age 11, which coincides with the start of secondary-schooling in the UK and has previously been used in research exploring generational differences in UK South Asians [20]. Participants were invited to a semi-structured, audio-recorded interview. We drew on the grounded theory methods described by Kathy Charmaz [21] to design the initial sample to be recruited, adapting this as we coded the interview transcripts and explored emergent themes.

### Data collection

We used a socio-ecological model to help develop the interview guide (S1 Table) to ensure we explored questions beyond the influence of ethnicity on physical activity. Questions were open-ended, covering: individual influences (attitudes to physical activity amongst their family, ethnic and religious group, impact of gender and age); social influences, such as the media, peers and colleagues’ physical environment influences, such as the local neighbourhood and the weather. Finally participants were invited to compare their experience of physical activity in childhood as opposed to now. During the interviews, participants were given a map of their local area to prompt discussions about how they use their neighbourhood for physical activity. Throughout the data collection process, the interviewer kept a field diary for noting immediate thoughts and summarising interviews. All interviews were conducted in English, although the interviewer understands Hindi and was therefore able to interview people who incorporated Hindi into their responses.

Recruitment used a variety of strategies devised after reviewing literature on recruiting minority groups into health research. We recruited people into the study by advertising in public spaces, email lists, approaching local community organisations, through primary schools and nurseries, and using snowballing techniques.

### Participants

This study was based in Greater Manchester, United Kingdom. We interviewed participants in locations spanning some of the most and least deprived areas in Greater Manchester. Area deprivation was measured using the Index of Multiple Deprivation [22]. We purposively sampled participants at different life stages and with varying occupations (Table 1). Participants had to be resident in Manchester/Greater Manchester to identify as having Indian ethnicity and at least 18 years old. Interviews were held in a location chosen by the participant and were conducted in cafes, libraries and people’s homes.

**Table 1:** Characteristics of first and second-generation participants.

		Second-generation (N = 19)	First-generation (N = 9)
**Age group**	20 to 29	9	0
	30 to 39	6	1
	40 to 49	3	0
	50 to 59	1	6
	60 to 69	0	2
**Religion**	Hindu	14	7
	Sikh	2	0
	Jain	1	2
	Muslim	2	0
**Family/personal**			
**migration**	East Africa	6	4
**history**	India	13	5
**Index of Multiple**	1 (most deprived)	6	1
**Deprivation**	2	6	2
**quintile**	3	2	1
	4	1	0
	5 (least deprived)	4	5
**Occupations as**	Account manager, advertising	1	0
**described by**	Administrator	0	1
**participants**	Bank Clerk	1	0
	Bank Portfolio Manager	1	0
	Clerical Officer	1	0
	Customer Services	1	1
	Dentist	1	0
	Doctor	1	2
	General assistant	0	1
	Retired bakery worker	0	1
	Software consultant	0	1
	Student	7	0
	Systems developer	0	1
	Teaching Assistant	2	1
	Trainee Solicitor	1	0
	Volunteer	1	0
	Works in family business	1	0

### Analysis

We analysed each interview as soon as it was transcribed, and continued to recruit participants until no new themes were emerging in the data. Decisions on which themes to explore in further detail with participants were made based on the findings from the previous interviews conducted. In the light of this, after the first ten interviews, we decided to include first-generation women in the study to provide an additional perspective on second-generation women’s views of generational change. First and second-generation interviews were analysed separately. Initially, interviews were coded using words or short phrases to summarise extracts. Constant comparative methods were used to explore similarities and differences between interviews. We then identified codes that were common across interviews and sorted them according to whether they related to a barrier, facilitator or a stage of life. PB reviewed all transcripts to ensure the initial codes used were appropriate and to check if any implicit or vaguely-stated concepts had been missed. Finally, we used theoretical codes to look for relationships and connections between codes and groups of codes. PB used memos to record insights into the codes and used these, along with the codes themselves, to develop understanding of their influences on physical activity. Once themes had emerged from the transcripts, we used the socio-ecological model as a general guide to group these themes. On top of this grouping, we identified which themes were facilitators and which were barriers to physical activity. What follows is a discussion of these themes according to whether they facilitate or impede physical activity among second-generation British Indian women; themes that emerged from the first-generation interviews are included where they elucidate differences that are important for public health interventions.

### Reflexivity

Acknowledging and understanding your own perspectives and influences as a research is key to conducting research in line with Grounded Theory, particularly so for Kathy Charmaz’ constructivist approach.

PB is a UK-born woman of Indian origin, whose ethnicity is likely to have influenced the data collection and analysis. Through sharing an ethnic background and gender with the participants, PB had personal experience of the ways in which physical activity has changed between generations in her own family, which influenced the research questions in this paper. Although we attempted to construct the interview guide using theory and existing evidence, the ethnic and gender identity of the interviewer is very likely to have influenced recruitment and the information provided by the participants. The interviewer grew up in Manchester and therefore was able to use some personal and family contacts to help with recruitment, although none of the participants were friends or family. Being of a similar ethnic background to the participants and being from Manchester may have helped some participants open up to the interviewer. For example, some people discussed the attitudes of White people or ‘Goras’ (the Hindi term for White people).

Participants may not have been as open about discussing the attitudes of White people in comparison to themselves if the researcher was not obviously from an ethnic minority. Being a woman may also have helped the women open up more, as there were discussions around clothing issues, expectations of women in the home and gender stereotyping of women. The most important issue was that the women trusted the researcher and that the women felt the topic was an important research area [23], with some women particularly expressing their opinion on importance of increasing physical activity in South Asian populations.

## Results

Four themes related to barriers: ways of socialising, physical appearance, safety concerns and prioritisation of education attainment. Six themes relating to facilitators in second-generation Indian women emerged from the data: importance of time for oneself, fewer domestic pressures, religion, physical education lessons, the media, local neighbourhood facilities; these cut across social and environmental factors (S2 Table). Here, we summarise these themes with illustrative quotes, highlighting generational differences where these emerged.

### Barriers

#### Ways of socialising

Many of the women discussed how most boys socialise through sport, for example, football, but most girls socialise in other, less-physical ways. As a result, the fact of being a girl meant that women were less active than their male peers during adolescence and adulthood. Nearly all participants who talked about this described this gender difference in how men and women socialise as being general and not specific to Indian people.

*“…there’s so many similar guys who have got similar ages in Manchester so that’s how they socialise*, *they just play football in the park*. *Whereas with girls it’s not like there’s an easy way to play a sport together"* (Participant 1, Second-generation).

#### Physical appearance

Almost all second-generation women felt motivated to take some form of exercise by pressure to look attractive or feminine, however there were also discussions of pressures to look ‘good’ during physical activity:

*“…going to the gym or going for a run and you bump into people*. *It’s not always like the nicest experience cos you’re not–you don’t look very nice do you*? *No make-up*, *you’re sweating everywhere*, *baggy t-shirt*, *then you see people and then they’re kind of like just*, *‘oh yeah*, *we saw you*, *you looked kind of a mess’*, *or I don’t know*, *I think it just puts you off in a way*, *like it throws you*.*”* (Participant 2, Second-generation)

One participant described having friends who were put off cycling because of anxieties about how they might look or mess up their hair, and another described feeling uncomfortably on display in the weights-area of a gym: “*we just used to get stared at…it just wasn’t a nice experience"* (Participant 2, Second-generation).

#### Safety concerns

Some second-generation women reported that their own outside play and active travel in childhood had been restricted by their parents’ concerns for girls’ safety: *“…they’d never feel comfortable sending me out by myself*, *so they’d drive me everywhere really”* (Participant 3, Second-generation). Participants were generally accepting of this and some did not mind that they had been restricted to some extent from playing outside as a child:

*“Compared to my male cousins*, *when I was growing up–there are like one or two who are older than me–I think they were allowed to like*, *go on the road and ride the bike and stuff like that*, *but we weren’t allowed to go and I knew that was because I was a girl so I think*, *but I didn’t stop me from–I don’t know how to describe it but I didn’t feel it was a bad thing that they were not letting us go cos I just thought like because they wanted me to be safe*. (Participant 1, second-generation)

#### Educational attainment expectations

In adolescence, education was often emphasised over other activities, meaning that physical exercise was side-lined for some participants when they were studying for GCSEs or A-levels.

*“… You’re going to get more in life by studying than going to the gym or playing sports*, *so (laughs)*. *That’s the way it was when I was growing up to be honest”* (Participant 6, Second-generation).

If participants were already seen to be doing well in school then physical activity was not discouraged, but some people reported that they thought they would have been discouraged from doing physical activities if they were not doing well at school.

*“*..*but I mean if we were*, *they would tell me*, *they’d be like ‘you need to do something’ not just to sit there*, *but I’d say when I got towards A-level*, *then they’d say it less*, *because they’d be like concentrate on your studies and less–like it’s not really that important*.*”* (Participant 4, second-generation)

### Facilitators

#### Importance of time for oneself

Second-generation women spoke about exercising a means of taking time out to do something for themselves. For instance, one participant described attending yoga classes.

*“…my husband goes to the gym*, *my kids go to the gym but I don’t do anything so that was just something for me"* (Participant 14, Second-generation).

This was in contrast to the first-generation, who were described, both by themselves and by the second-generation, as prioritising the family over themselves.

*“…because they [mothers] have to take care of*, *like they don’t put themselves first*, *do they*? *Don’t take care of their health so they don’t do exercise*, *like*, *they think about oh they’ve got their in-laws and then their husband and then their kids*, *so they’ll put all of their priorities first and put them[selves] last*. (Participant 20, Second-generation)

#### Fewer domestic pressures

Most participants noted that girls in Indian families are expected to do more housework than boys, and that they themselves, as children, had done more housework than their brothers. The generations differed, however, regarding expectations of the wife and mother to look after the family. First-generation women described being encouraged as children by their parents to do the housework, because they would be expected to maintain the household when they married, and spoke about prioritising family care and cooking above all other activities except housework.

*“So for Indians I know that even though I was studying in medicine and it was a very heavy study commitment*, *my mum was making sure that I have to get up at 5 o clock in the morning and help rolling chapatti for the day so that I don’t forget*, *so that when I get married that I don’t have problem…”* (Participant 24, First-generation)

For the second-generation, family responsibility was less of a concern, especially for women in the 20 to 24 age group who did not have children. Among women who had children, or were married, the second-generation were more likely to prioritise their physical activity:

*“we haven’t got that burden on us that much*, *so we can focus on us”* (Participant 20, Second-generation).

#### Religion

Second-generation Hindus, Sikhs and Jains generally did not believe their religion influenced their decisions about exercise, though there were some exceptions. Hindu women who said their religion does influence their exercise cited yoga and ‘mental exercises’, but were unclear about the precise role of yoga in Hinduism. Some women discussed the role of religious clubs in encouraging physical activity, rather than the religion itself.

*“…we were part of a Hindu group–it was a Hindu group called Shaka and then*, *so from the age of when I was about 10 or so*, *this group actually encouraged us to actually participate in sport*, *so we did marathons and things like that*, *you know*, *fun runs*?*”* (Participant 6, Second-generation)

Muslim women cited the Koran, saying that its health messages were similar to the health messages they have heard in other media sources. The few Muslim women in the study commented that Islam influences how women exercise by prescribing modesty and non-mixing with men, but this was not necessarily described as a barrier, just as a prescription to the way they should exercise.

“*Well–it [the Qur’an] does say you should exercise but in a modest way*. *Like Islam would be against*, *like you can go swimming*, *but then it would tell you*, *you can’t just go out in a bikini or a swimming costume*, *so you can go swimming*, *but modestly you know*? *Cover yourself*, *because we have Islamic swimming costumes*, *where can cover you from top to toe*.*”* (Participant 19, Second-generation)

#### Physical education (PE) lessons in school

Participants who had completed all or at least part of their schooling in the UK described positive experiences of doing sports and PE in school. Second-generation women said that although they were not encouraged to exercise at home, this was sometimes because their parents believed that their daughter was getting exercise through school PE. Many second-generation women discussed the positive impact of PE at school in teaching them about the importance of physical activity. This was true even of women who described themselves as ‘not sporty’ or not in the school sports teams:

*“I think we’ve learnt through school and stuff–we’ve been told how important exercise is and to have an active lifestyle is good for your health” (Participant 7, Second-generation)*.

No first-generation participant reported that their school had directly addressed this issue, even if they had done sports at school.

#### The media

Second-generation women reported that mainstream English-language media (television, films, magazines and the news) provide a general message that activity is important, though this is usually about weight and looks, rather than physical activity for health.

*“I don’t think they [the media] say anything about the amount you should be exercising*, *just that you should be thin*.*”* (Participant 4, Second-generation)

The majority of first-generation participants watched Asian television channels almost exclusively, and described these channels as having no physical activity message, with the exception of yoga. The second-generation reinforced these observations this when talking about their parents:

*“My dad watches Indian channels*, *which is where I could see it*, *but as I say*, *those channels come from India and they don’t really push health as much as we do here*.*”* (Participant 4, Second-generation).

#### Local neighbourhood facilities for physical activity

Both generations were aware of local facilities in their area, such as parks and leisure centres. Some second-generation women described using local parks to socialise in, or to run in. By contrast, almost all of the first-generation women described parks as being for children, and said they would only go there with children:

*“When they were young children I used to go*, *but now I don’t go park*.*”* (Participant 12, First-generation).

Some of the second-generation also described only going to parks with children, but this attitude was not universal.

## Discussion

Barriers and facilitators to physical activity among second-generation Indian women span social and environmental factors. Ways of socialising, concerns over appearance while being physically active, safety concerns and prioritising educational attainment in adolescence were the cited barriers; the first three barriers listed here have all been reported as barriers in studies on women in general, rather than on British Indian women specifically. Facilitators for physical activity that emerged from the data included acknowledging the importance of taking time out for oneself; religious beliefs and religious groups promoting activity; being prompted by family illness; positive messages in both the media and while at school, and having local facilities to use.

Our finding that British Indian women reported socialising through activities that are not physically active has been described in literature that is focused on a general population. A focus group study of teenage girls in South Australia reported that the girls would rather go shopping or do other activities than play sports; this is in contrast to boys who socialised through sports [24]. This indicates that gender differences in ways of socialising may affect the physical activity levels of girls regardless of ethnic group.

Having concerns over appearance during physical activity has previously been reported among young women and teenage girls in English-speaking countries (the UK, the United States, and Australia). These studies all report that women and teenage girls in these countries worry about sweating during exercise and about maintaining a ’feminine’ appearance while playing sports, implying that that participating in physical activity conflicts with their ideas of feminine identity [24–29]. These observations resonate with comments made by participants in our study about how some women feel pressure to look good while doing physical activity, with some succumbing to this and others being active regardless. Maintenance of a ’feminine’ appearance was also a motivator for some women in our study, which is line with literature exploring motivations for physical activity in the general population in the UK [30].

Heightened safety concerns as a barrier to active travel is a barrier reported by women in the general population, as well as by people of South Asian ethnicity. Focus groups carried out with women in Glasgow describe fear for personal safety when walking or cycling in the dark, or fear of being harassed [31]. In our study, this fear was mainly described as being a barrier in childhood as parents feared for their daughter’s safety and therefore tended to drive their children to destinations. It is might be that restrictions on active travel in childhood track through to adult commuting behaviours because the habit of active travel was not formed.

Some women in our study reported that education was prioritised over physical activity during their childhood. Platt (2005) suggests that the high levels of achievement of second generation British Indians and African Asians may be due to their migrant parents asserting their original background through their children, by encouraging education and emphasising its importance [32]. Abbas (2002) interviewed parents and children of South Asians in Birmingham [33] and discusses how differences in parents’ social capital, knowledge and religion influence the education experiences of South Asians in Birmingham.

The second-generation women in our study reported using the green spaces in their areas, which was in contrast to the first-generation. Differential use of green spaces has been previously reported in a review on outdoor recreation and ethnicity in Europe [34]. Interestingly Gentin (2011) reports mixed findings where Pakistani teenagers report being comfortable in parks in Sheffield, whereas an on-site survey of parks in Leicester indicated that South Asian and African-Caribbean groups were afraid of racial attacks. It is possible that these mixed findings are due to the differing generational statuses of the participants in the two studies, although this is not reported. This research also points to a possibility that one of the reasons the first-generation did not use parks once their children were grown was due to fear of racism, although no participant discussed this.

Our findings indicate that faith-based settings could be utilised to promote physical activity among some British Indian women. A recent scoping review examining health promotion interventions for obesity in Islamic religious settings found that there is some evidence that physical activity interventions in these settings are worthwhile [35]. A study exploring using Christian, Hindu and Sikh settings in London for obesity interventions however struggled with recruitment [36]. A study using faith-based settings for recruitment of South Asians for screening for a trial, rather than for delivering the intervention itself had more success in recruiting participants [37], therefore more research is needed in this area.

Media messaging and positive school messaging are part of a wider trend of physical activity being a social norm. Some of the women in our study described how, when their parents provided no opinion on physical activity, the education they received in schools served to provide a positive one; when participants had been encouraged to be active by their parents, school education reinforced these messages. Curriculum changes during the late 20^th^ Century allowed for more choice of activities and encouraged activities outside of school time. In conjunction with the increase in sports and leisure provision in local areas from the 1970s onwards, these changes will have increased both the opportunity and aspiration to do physical activity [38]. It is likely that physical education classes, while perhaps promoting particular sports, particularly for girls [39], has had an overall positive impact on the attitudes towards physical activity in the second-generation Indian women in our study.

### Implications

Culturally tailored interventions may still be useful for second generation Indian women, especially if targeting specific religious groups and if being aimed at adolescents. However, family-level interventions may need a different approach that takes into account the fact that attitudes towards domestic priorities differ between the generations. As some of the barriers and facilitators described by second-generation Indian women are similar to those described by women in the general population, new and existing public health interventions aimed at the women in the general population may effective.

### Conclusions

Barriers and facilitators to physical activity in second-generation British Indian women have differences from the first-generation that may impact cultural tailoring. Interventions aimed at women in the general population may be more effective for the second-generation than the first generation. The findings from this paper may not be transferable to some British Pakistani or Bangladeshi groups, which are more likely to have lower incomes, live in more deprived areas and follow Islam. Future research should therefore explore the impact of religion further and include more women on lower incomes. The factors influencing physical activity are likely to vary for non-South Asian British ethnic groups and future research should also examine potential intergenerational differences in these influences.

## Supporting information

S1 Table Topics included in interview guide. (DOCX)

S2 Table Emergent themes with place in socioecological model and illustrative quotes. (DOCX)
