# Supplementary material for: Barriers and facilitators to physical activity in second-generation British Indian women: A qualitative study
Source: PLoS One. 2021 Nov 3;16(11):e0259248. doi: 10.1371/journal.pone.0259248 (PMC8565737; doi:10.1371/journal.pone.0259248)
Supplement: S1 Table — (DOCX) [file pone.0259248.s001.docx]

**Supplementary table 1 Topics included in interview guide**

| **Overall theme/main question** | **Sub-theme** | **Further breakdown of sub-theme** |
| --- | --- | --- |
| Clarification will be talking about the activity do during your free- time, while walking, and while in the home. |  |  |
| How active would you say you are? |  |  |
| Individual influences on physical activity | Ethnicity | - Parental attitudes - Parental behaviour - Wider family attitudes - Others in ethnic group - Religion |
|  | Gender |  |
|  | Age group |  |
|  | Personal interests |  |
| Social environment’s influence on activity | Media |  |
|  | Colleagues |  |
|  | Friends |  |
| Physical environment’s influence on activity | Local neighbourhood |  |
|  | Area around work |  |
|  | Climate and weather |  |
|  | Drawing of neighbourhood map | Would you mind marking out on this map where your neighbourhood is? There is no right or wrong answer, I’m just looking to see which areas we have been discussing. |
| Exercise now and in childhood |  |  |
| How important do you think exercise is? |  |  |
|  |  |  |
