# Supplementary material for: Barriers and facilitators to physical activity in second-generation British Indian women: A qualitative study
Source: PLoS One. 2021 Nov 3;16(11):e0259248. doi: 10.1371/journal.pone.0259248 (PMC8565737; doi:10.1371/journal.pone.0259248)
Supplement: S2 Table — (DOCX) [file pone.0259248.s002.docx]

**Supplementary Table 2 Emergent themes with place in socioecological model and illustrative quotes**

|  | **Theme** | **Socioecological model level** | **Illustrative quote** |
| --- | --- | --- | --- |
| **Barriers** | Ways of socialising | Social | Yeah, it’s not just exercise, it’s stuff like, you know, playing football 'cos there’s so many similar guys who have got similar ages in Manchester so that’s how they socialise, they just play football in the park. Whereas with girls it’s not like there’s an easy way to play a sport together (Participant 1, Second-generation, Trainee solicitor). |
|  | Physical appearance | Individual | When I used to go to the gym quite a lot last year we used to go to like the weights because obviously not a lot of women do, but it’s actually the quickest way to lose weight in fact, so we used to go and we just used to get stared at…it just wasn’t a nice experience, so like you always had to go somewhere else and maybe not do weights when other people are there. (Participant 2, second-generation, age 22, Medical student)  I think ladies over here ... English born-brought up, they are quite into it ... I’m talking about British – they are quite active... totally into exercising, but then their culture is totally different. …. They come back home and again go for a run – [exclaims] aii – we don’t do all those things, we don’t make that effort to maintain ourselves. I think they have activity in the culture because they want to look good in pubs and they have all this culture isn’t it? Going for holidays to Spain, they have to look glamorous. We’re not bothered, we don’t bother about our looks – once we get married, I don’t think so anybody is bothered about our looks. And they say that openly as well. Abhi kya karna abhi? [What do you need to do now?] So you think this is not important, and anyway if I maintain myself there’s no point. It’s my husband who will be looking at me, so it’s ok [laughs] (Participant 9, First-generation, Software consultant). |
|  | Safety concerns | Social | ... I do more walking here [at university] 'cos when I was at home, and because you’re younger, then ... my parents would never really send me out to walk anywhere really. When I was in year 7, or year 6 age, I did Brownies and ... the place I did it was only like a 10 minute walk away, but they’d never feel comfortable sending me out by myself, so they’d drive me everywhere really (Participant 3, Second-generation, Medical student).  Compared to my male cousins, when I was growing up – there are like one or two who are older than me – I think they were allowed to like, go on the road and ride the bike and stuff like that, but we weren’t allowed to go and I knew that was because I was a girl so I think, but I didn’t stop me from – I don’t know how to describe it but I didn’t feel it was a bad thing that they were not letting us go cos I just thought like because they wanted me to be safe. (Participant 1, second-generation, Trainee solicitor) |
|  | Educational attainment expectations | Social | … You’re going to get more in life by studying than going to the gym or playing sports, so (laughs). That’s the way it was when I was growing up to be honest (Participant 6, Second-generation, Dentist). |
| **Facilitators** | Time for oneself | Individual | I used to go there a few years ago and then stopped for a while, and then started back again [because] I think it’s more doing something for myself, having time for myself to do something, because I don’t really do anything out on a regular basis. Like my husband goes to the gym, my kids go to the gym but I don’t do anything so that was just something for me... It gets me out with my friends as well, and I think we all enjoy it. Because we all go together, we all car-share, it’s just fun (Participant 14, Second-generation, Bank clerk). |
|  | Fewer domestic pressures | Social | “[In the]…morning when my daughter would [wakes up], even having a baby just now – very young baby – first thing she’ll ask me, ‘mummy is it ok if I go on the treadmill?’ At the time when I would be doing my exercise when I say exercise – the work in the kitchen – getting the dough ready which my muscles are working making the dough while her priority is, 'every day I have to go on treadmill'…” (Participant 24, First-generation, Doctor)  I think our generation – I think we still worry about it less, I think, I think for us because we don’t really think about maybe the extended family, it’s only just [us] and our husband and then our children. We don’t have to take care of all the others, and then usually our husbands are quite independent as well, so I think we haven’t got that burden on us that much, so we can focus on us. (Participant 20, second-generation, Works in family business) |
|  | Religion | Social | – Islam would say go to a ladies’ gym. You can go to the gym, there’s no harm in it, but go to the ladies’ gym. Islam doesn’t like it when you mix with other men – do you get it? (Participant 19, Second-generation, Teaching assistant). |
|  | Physical education lessons in school | School environment | I think like, we’ve like learnt from school recently, like everyone was pushed to exercise and like the Olympics and everything – the health benefits, but I don’t know if she thinks it’s that important. I think we’ve learnt through school and stuff – we’ve been told how important exercise is and to have an active lifestyle is good for your health, but I don’t know if she’s had – I don’t think she realises that it’s probably good for her to do more exercise (Participant 7, Second-generation, Mathematics student). |
|  | The media | Commercial environment | My dad watches Indian channels, which is where I could see it, but as I say, those channels come from India and they don’t really push health as much as we do here. It never really comes up, like the people on the screen will be thin but it’s not pushed. Like if you pick up a magazine here it’s like, lose this many pounds in a week (Participant 4, Second-generation, Medical student). |
|  | Local facilities for physical activity | Physical environment | PB: Why don’t you go to the park?  When children were young that time I used to go play with children, they liked to go park, very young children, that time I used to go there. That place swinging and just running round with the park, everything, they like. When they were young children I used to go, but now I don’t go park. (Participant 12, First-generation, Supermarket General Assistant). |
